# Association of facial ageing with DNA methylation and epigenetic age predictions

**DOI:** 10.1186/s13148-018-0572-2

**Published:** 2018-11-08

**Authors:** Riccardo E. Marioni, Daniel W. Belsky, Ian J. Deary, Wolfgang Wagner

**Affiliations:** 1 Centre for Genomic and Experimental Medicine, Institute of Genetics and Molecular Medicine, University of Edinburgh, Edinburgh, EH4 2XU UK; 2 Centre for Cognitive Ageing and Cognitive Epidemiology, University of Edinburgh, Edinburgh, EH8 9JZ UK; 3 Department of Population Health Sciences, School of Medicine, Duke University, Durham, NC 27710 USA; 4 Social Science Research Institute, Duke University, Durham, NC 27710 USA; 5 Department of Psychology, University of Edinburgh, Edinburgh, EH8 9JZ UK; 6 Helmholtz-Institute for Biomedical Engineering, Stem Cell Biology and Cellular Engineering, RWTH Aachen Faculty of Medicine, Aachen, Germany

**Keywords:** DNA methylation, Facial ageing, Epigenetic clock, Survival

## Abstract

Evaluation of biological age, as opposed to chronological age, is of high relevance for interventions to increase healthy ageing. Highly reproducible age-associated DNA methylation (DNAm) changes can be integrated into algorithms for epigenetic age predictions. These predictors have mostly been trained to correlate with chronological age, but they are also indicative for biological ageing. For example, accelerated epigenetic age of blood is associated with higher risk of all-cause mortality in later life. The perceived age of facial images (face-age) is also associated with all-cause mortality and other ageing-associated traits. In this study, we therefore tested the hypothesis that an epigenetic predictor for biological age might be trained on face-age as a surrogate for biological age rather than on chronological age. Our data demonstrate that facial ageing does not correlate with either the epigenetic clock or blood-based DNAm measures.

**Electronic supplementary material:**

The online version of this article (10.1186/s13148-018-0572-2) contains supplementary material, which is available to authorized users.

## Main text

We analysed data from the Lothian Birth Cohort 1921 (LBC1921), a longitudinal study of ageing in a 1921 birth cohort followed up at five assessment waves between ages 79 and 92 years (Additional file 1) [1]. DNA methylation profiles were analysed in whole blood collected at mean age 79.1 (SD 0.55) years using Illumina HumanMethylation450BeadChips as previously described [2]. Perceived facial age (face-age) was assessed from neutral expression facial photographs taken at mean age 83.3 years (SD 0.52; blood samples were not collected at this time point) [3]. Briefly, 12 university students (6 male, 6 female) estimated the participants ages based on high-resolution photographs, taken under the same lighting conditions, at the same distance, using the same camera. The images were presented one at a time on a high-quality cathode ray tube computer monitor. Face-age acceleration was calculated as the (linear regression) residuals of face-age regressed on chronological age. Mean estimated face-age was 74.2 years (SD 3.9, range 63.5–85.3). The face-ages that raters assigned LBC1921 participants based on their photographs tended to be younger than their true chronological ages when the photographs were taken. This could reflect LBC1921 being a relatively healthy and long-lived cohort. By the time they were enrolled at age 79 years, these individuals had outlived their birth cohort’s life expectancy by more than two decades [4]. Because our analysis depends on relative differences between estimated facial ages, whether the average face-age tends to be older or younger than true chronological age will not affect our results. Overall, DNAm measurements and face-age assessments were available for 235 individuals (43% female, 6% current smokers, 49% ever smokers).

**Fig. 1:**
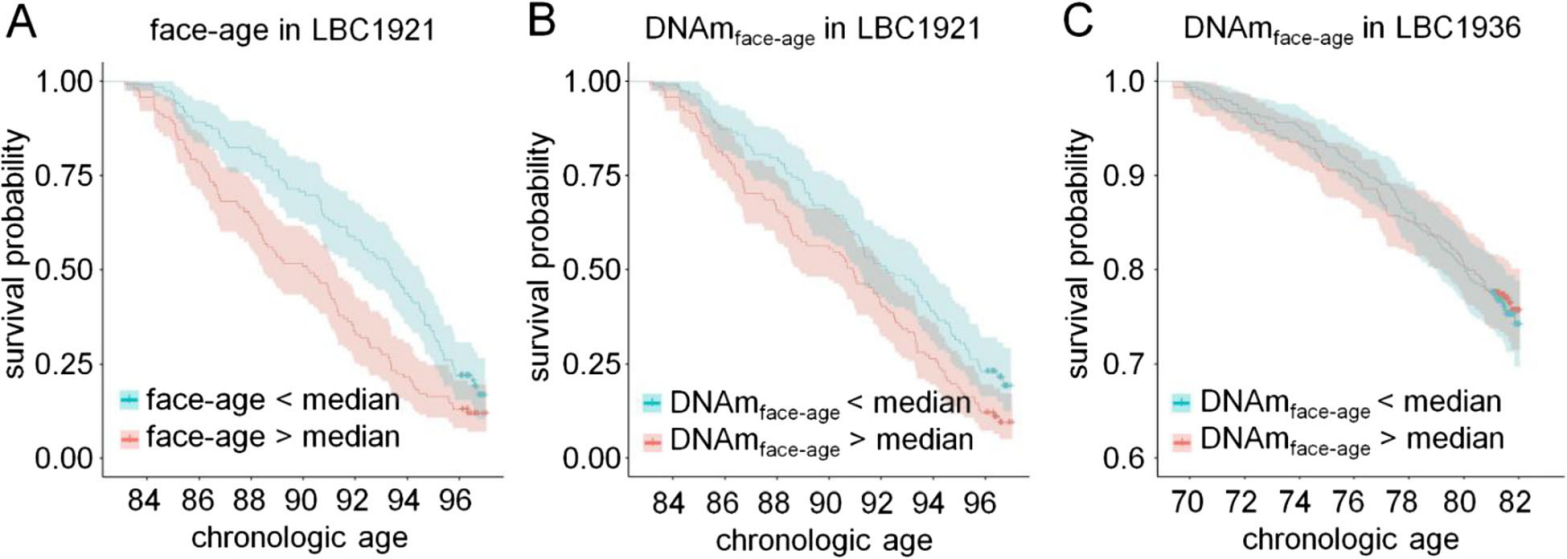
Perceived facial ageing is associated with all-cause mortality, but not with DNA methylation signatures trained on perceived ageing. **a** Kaplan-Meier plots depict survival rates of LBC1921 participants stratified by the median perceived age in facial images (face-age). **b** Alternatively, the participants were stratified by mean age predictions based on an algorithm of 32 CpGs that was trained on face-age of the LBC1921 (DNAm_face-age_). **c** The results with this algorithm did not replicate in the independent LBC1936 cohort

Perceived face-age has been linked to mortality risk [5] and other ageing-associated traits [6]. The relationship between older face-age and increased mortality risk was also evident in a previous data release of LBC1921 [3], and again here using updated survival information (HR 1.39 [1.19, 1.63] per SD increase in face-age, *p* = 3.5 × 10^− 5^; Fig. 1a). People with older face-age also show signs of accelerated biological ageing as measured from physiology-based indices (Pearson *r* ~ 0.2) [7]. Evidence for association of older face-age with epigenetic ageing is more sparse; in midlife adults of the Dunedin study, there was a small effect-size association with one epigenetic clock but no association with two others [7]. We therefore first tested associations between face-age and epigenetic age predictors proposed by Horvath (based on 353 CG dinucleotides - CpGs) [8], and two signatures that were trained on blood samples by Hannum et al. (71 CpGs) [9] and Weidner et al. (99 CpGs) [10]. In the LBC1921 cohort, consisting of older adults, accelerated epigenetic ageing (residual of epigenetic age regressed on chronological age) was not associated with higher face-age (*r*_Horvath:face-age_ = 0.06, *P* = 0.35; *r*_Hannum:face-age_ = 0.01, *P* = 0.93; and *r*_Weidner:face-age_ = 0.01, *P* = 0.82).

We then tested the associations between face-age and DNAm of 307,745 individual CpGs (Additional file 1 and Additional file 2: Table S1). The maximum absolute Spearman correlation of face-age with DNAm was *r* = 0.29 (*P* = 5.1 × 10^−6^) for cg18402261. To take potential confounding effects into account, we then performed epigenome-wide association study (EWAS) analysis with three nested regression models: the base model (M1; Additional file 2: Table S2) included covariates for age, sex, and technical factors (plate, array, position, hybridisation date); additional models added covariates for smoking history (M2; Additional file 2: Table S3) and measured white-blood-cell counts (neutrophils, lymphocytes, monocytes, eosinophils, and basophils; M3; Additional file 2: Table S4). The lead CpG from the most conservative model (M3) was cg00871706 (*P* = 1.8 × 10^−6^, chr7:138,666,647, *KIAA1549*). Pathway analysis of the closest genes to the top 100 CpGs from this most conservatively modelled EWAS identified no evidence of functional enrichment (Bonferroni *P* > 0.05). Taken together, the perceived age based on facial photographs revealed no genome-wide significant associations with DNAm at specific CpG sites or with gene sets linked to specific biological pathways.

Finally, we tested if a linear combination of CpGs might provide information about biological ageing. We conducted LASSO regression analysis on all 307,745 CpGs although this returned only an intercept term and single CpG site in the predictor (Additional file 1). We therefore restricted the penalised regression input to the top 100 CpGs in the M3 EWAS to facial age. The resulting algorithm included 32 CpGs (Additional file 2: Table S5). In the LBC1921 training sample (*n* = 235, *n*_deaths_ = 198), the epigenetic predictor of facial age was correlated with measured facial age (*r* = 0.66) and predicted increased risk for mortality (HR 1.31 [1.12, 1.53] per SD increase in face-age, *p* = 8.2 × 10^−4^; Fig. 1b). The association with mortality risk was in the same direction but had a much smaller effect-size and was not statistically significant in the out-of-sample analysis in the independent Lothian Birth Cohort 1936 (*n* = 920, *n*_deaths_ = 215; HR 1.07 [0.94, 1.23], *p* = 0.32; Additional file 1; Fig. 1c).

This is the first study on epigenome-wide association of DNAm with perceived facial age. Face-age was clearly associated with all-cause mortality in the 235 LBC1921 individuals, as previously reported [3]. However, our results did not support the hypothesis that an epigenetic measure of biological age could be derived from an analysis of facial ageing. Efforts to train epigenetic predictors of biological ageing on surrogate biological ageing measures, such as face-age, may require larger sample numbers. Precise epigenetic ageing signatures have been trained to predict chronological age in fewer samples [10]. The need for more samples to train DNA methylation algorithms that predict face-age could reflect a greater degree of measurement error in face-age as a criterion or simply differences in the degree to which DNA methylation reflects variation in this phenotype. It is also possible that the time of blood collection at the age of 79 and 70 (for LBC 1921 and LBC 1936, respectively) is not ideal for such correlative analysis. In fact, age-related DNAm changes in peripheral blood occur more rapidly during childhood and the progression of facial ageing might differ in younger individuals, too. Longitudinal data and cohorts with a wide age-range would help to determine the temporal relationships of face-age and DNAm. A second limitation is that face-age and DNAm were measured in the skin and blood, respectively. It is well known that age-associated modifications occur in both tissues [8]. But the pace of biological ageing may vary across different tissues. DNAm analysis in the skin should therefore be a priority in future face-age analyses; however, such datasets are not yet available. Similarly, and to our knowledge, there are no existing, appropriately aged datasets available with data on face-age, DNA methylation, and survival, which would allow replication of our findings. Our results indicate that face-age is uncorrelated with both blood-based individual CpG levels and epigenetic clock estimates, despite face-age itself being proposed as a measure of biological age.

## Additional files

Additional file 1: Data from the Lothian Birth Cohort 1921 (LBC1921). (DOCX 13 kb)

Additional file 2: **Table S1**: Descriptive information for the 1000 most correlated CpGs with face-age. **Table S2**: Top 1000 face-age-CpG associations from EWAS model 1 . **Table S3**: Top 1000 face-age-CpG associations from EWAS model 2. **Table S4**: Top 1000 face-age-CpG associations from EWAS model 3. **Table S5**: 32 CpG predictor of face-age built from the top 100 CpGs reported in **Table S4**. (XLSX 240 kb)

## Abbreviations

DNAm: DNA methylation
Face-age: Perceived facial age
LBC: Lothian Birth Cohort

## References

[1] Taylor AM, Pattie A, Deary IJ. Cohort profile update: the Lothian Birth Cohorts of 1921 and 1936. Int J Epidemiol. 2018;47(4):1042–1042r. 10.1093/ije/dyy022 PMC6124629 29546429

[2] Marioni RE, Shah S, McRae AF, Chen BH, Colicino E, Harris SE (2015). DNA methylation age of blood predicts all-cause mortality in later life. Genome Biol.

[3] Dykiert D, Bates TC, Gow AJ, Penke L, Starr JM, Deary IJ (2012). Predicting mortality from human faces. Psychosom Med.

[4] National Records of Scotland. Life tables for Scotland 2014–2016 [Internet]. [Accessed 2018 Sep 19]. Available from: https://www.nrscotland.gov.uk/statistics-and-data/statistics/statistics-by-theme/life-expectancy/life-expectancy-at-scotland-level/scottish-national-life-tables/2014-2016/national-life-tables

[5] Christensen K, Thinggaard M, McGue M, Rexbye H, Hjelmborg J v B, Aviv A (2009). Perceived age as clinically useful biomarker of ageing: cohort study. BMJ.

[6] Shalev I, Caspi A, Ambler A, Belsky DW, Chapple S, Cohen HJ (2014). Perinatal complications and aging indicators by midlife. Pediatrics.

[7] Belsky DW, Moffitt TE, Cohen AA, Corcoran DL, Levine ME, Prinz JA, et al. Eleven telomere, epigenetic clock, and biomarker-composite quantifications of biological aging: do they measure the same thing? Am J Epidemiol. 2018;187(6):1220–1230. 10.1093/aje/kwx346 PMC6248475 29149257

[8] Horvath S (2013). DNA methylation age of human tissues and cell types. Genome Biol.

[9] Hannum G, Guinney J, Zhao L, Zhang L, Hughes G, Sadda S (2013). Genome-wide methylation profiles reveal quantitative views of human aging rates. Mol Cell.

[10] Weidner CI, Lin Q, Koch CM, Eisele L, Beier F, Ziegler P (2014). Aging of blood can be tracked by DNA methylation changes at just three CpG sites. Genome Biol.

