# Supplementary material for: Association of facial ageing with DNA methylation and epigenetic age predictions
Source: Clin Epigenetics. 2018 Nov 8;10:140. doi: 10.1186/s13148-018-0572-2 (PMC6225560; doi:10.1186/s13148-018-0572-2)
Supplement: Supplementary file 1 — Data from the Lothian Birth Cohort 1921 (LBC1921). (DOCX 13 kb) [file 13148_2018_572_MOESM1_ESM.docx]

# Additional file 1

## The Lothian Birth Cohorts

The Lothian Birth Cohort 1921 (LBC1921) is a longitudinal study of aging [1]. All participants were born in 1921 and have been followed up every few years from ages 79 to 92 years, yielding a maximum of 5 waves of data per participant. At each wave, cognitive, personality, health and disease data were collected.

The Lothian Birth Cohort of 1936 (LBC1936) includes participants that were born in 1936 and have been followed up every three years from ages 70 to 82 years [1]. The most recent wave of data collection at age 82 years is currently ongoing. As with the LBC1921 study, cognitive, personality, health and disease data were collected at each wave.

Survival data are routinely collected in LBC via data linkage to the Scottish National Health Service Central Register. Data for this study were correct as of January 2018.

## DNA methylation

DNA methylation data were assessed in whole blood samples from the LBC studies using the Illumina HumanMethylation450 BeadChip (Illumina Inc., San Diego, CA). Data from the first wave of data collection in both LBC studies were considered for the current analysis: LBC1921 participants were a mean age of 79.1 years (SD 0.55 years); LBC1936 participants were a mean age of 69.5 years (SD 0.83 years). Background correction was performed and quality control was used to remove probes with a low detection rate (P>0.01 for >5% of samples), low quality (manual inspection), low call rate (P<0.01 for <95% of probes), and samples with a poor match between genotypes and SNP control probes, with incorrectly predicted sex. This left a dataset of 450,727 CpGs, which was further filtered to exclude X and Y, cross-reactive, non-cg, and SNP-in-probe-sequence CpGs, The final EWAS dataset therefore consisted of 307,745 CpGs.

## Face-age and DNAm age acceleration correlations

Spearman correlations were computed between the age acceleration measures (face-age from age 83 years and DNAm age from age 79 years).

## Epigenome-wide association studies

Three linear regression models were considered for the EWAS as described in the text. The DNAm values at individual CpGs were the dependent variables and face-age was the dependent variable of interest. A Bonferroni correction was applied to account for multiple testing (P<0.05/307,745=1.6x10^-7^).

## Functional enrichment

The top 100 CpGs from the most conservative EWAS model (M3) were examined for functional enrichment (closest gene to the nearest transcription start site was taken as the input) based on a PANTHER Gene Ontology analysis with Bonferroni P<0.05 set as the significance threshold.

## LASSO regression

A parsimonious predictor of face-age was then generated via least absolute shrinkage and selection operator (LASSO) regression using the ‘glmnet’ package in R. Mean imputation was used for any missing CpG values. 10-fold cross-validation was applied with the mixing parameter (alpha) set to 1 (LASSO penalty). Coefficients were extracted for the model with the minimum mean cross-validated error estimate. A model including all 307,745 CpG sites as features returned a best-fit predictor with only an intercept and single CpG site (cg05071974). The CpG site correlated -0.08 with face-age in the training dataset, which is well below the highest observed correlation of 0.29, implying a very poor fit. We restricted the feature selection to the top 100 CpGs most associated with face-age from the M3 EWAS and re-ran the LASSO model. However, this approach is biased, particularly for the prediction back into LBC1921, as it estimates the penalised regression weights from a pre-selected set of CpGs that are known to correlate with face-age in the training dataset.

Age- and sex-adjusted Cox proportional hazards models with DNAm-based face-age as the predictor and time-to-event (death or censoring) as the outcome were performed in the training and test datasets (LBC1921 and LBC1936, respectively). Analyses were conducted in R using the ‘survival’ package.
